# Expanding the Phenotypic and Genotypic Spectrum of *ARFGEF1*-Related Neurodevelopmental Disorder

**DOI:** 10.3389/fnmol.2022.862096

**Published:** 2022-06-17

**Authors:** Lu Xu, Youfeng Zhou, Xiaoyan Ren, Chenlu Xu, Rongna Ren, Xuke Yan, Xuelian Li, Huimin Yang, Xuebin Xu, Xiaotong Guo, Guoxia Sheng, Yi Hua, Zhefeng Yuan, Shugang Wang, Weiyue Gu, Dan Sun, Feng Gao

**Affiliations:** ^1^Department of Neurology, National Clinical Research Centre for Child Health, The Children's Hospital, Zhejiang University School of Medicine, Hangzhou, China; ^2^Department of Pediatrics, Fujian Provincial Maternity and Children's Hospital, Affiliated Hospital of Fujian Medical University, Fuzhou, China; ^3^Beijing Chigene Translational Medical Research Centre Co. Ltd., Beijing, China; ^4^Department of Pediatrics and Neurosurgery, 900 Hospital of the Joint Logistics Team, Fuzhou, China; ^5^Department of Pediatric Neurology, Anhui Provincial Children's Hospital, Hefei, China; ^6^Department of Pediatric, Inner Mongolia Maternal and Child Health Care Hospital, Hohhot, China; ^7^Department of Pediatric Neurology, Wuhan Children's Hospital, Tongji Medical College, Huazhong University of Science and Technology, Wuhan, China

**Keywords:** *ARFGEF1*, mutation spectrum, data lake, whole-exome sequencing, neurodevelopmental delay

## Abstract

Mono-allelic loss-of-function variants in *ARFGEF1* have recently caused a developmental delay, intellectual disability, and epilepsy, with varying clinical expressivity. However, given the clinical heterogeneity and low-penetrance mutations of *ARFGEF1*-related neurodevelopmental disorder, the robustness of the gene-disease association requires additional evidence. In this study, five novel heterozygous *ARFGEF1* variants were identified in five unrelated pediatric patients with neurodevelopmental disorders, including one missense change (c.3539T>G), two canonical splice site variants (c.917-1G>T, c.2850+2T>A), and two frameshift (c.2923_c.2924delCT, c.4951delG) mutations resulting in truncation of ARFGEF1. The pathogenic/likely pathogenic variants presented here will be highly beneficial to patients undergoing genetic testing in the future by providing an expanded reference list of disease-causing variants.

## Background

The *ARFGEF1* (MIM 604141, previously known as *BIG1*) is a 39-exon gene that maps to the 8q13 locus on a chromosome and is highly conserved in mammals and eukaryotes (Wright and Kahn, 2014). *ARFGEF1* encodes a 209-kDa protein that participates in the ADP-ribosylation factors (ARFs) activation by accelerating the replacement of bound GDP with GTP (Cherfils et al., 1998). *ARFGEF1* proteins feature a Sec7 domain, which may be responsible for their guanine-nucleotide exchange activity and brefeldin A inhibition. Aside from the Sec7 domain, additional highly conserved domains and sequences in *ARFGEF1* are less functionally defined (Bui et al., 2009). ARFGEF1 is necessary for Golgi integrity, mature integrin β1 glycosylation (Shen et al., 2007), and neurite development (Zhou et al., 2013).

*ARFGEF1, ARFGEF2* (MIM 605371), and *ARFGEF3* (MIM 617411) are members of the BIG/GBF1 family in humans. *ARFGEF2* mutations are associated with autosomal recessive periventricular nodular heterotopia with microcephaly (Banne et al., 2013; MIM 608097). *ARFGEF1* is related to genetic epilepsy in linkage and association studies (Wallace et al., 1996; Piro et al., 2011; Addis et al., 2018). Teoh et al. (2019) reported an *ARFGEF1* nonsense variant in a patient with Lennox–Gastaut syndrome. Another recent study revealed *ARFGEF1* heterozygous truncating variants in 13 patients from 11 families with a novel developmental delay condition caused by haploinsufficiency (Thomas et al., 2021; without a MIM# assigned at the time of manuscript preparation).

Neurodevelopmental disorders are quite prevalent, affecting an estimated 1–3% of the population (Mefford et al., 2012). While diverse mechanisms, including gestational infection and maternal alcohol consumption, can cause such neurodevelopmental disorders, damaging genetic variation of essential genes during neurodevelopment is one of them. Therefore, understanding the genetic factors underlying neurodevelopmental disorders is critical. Rare variants in hundreds of genes have been implicated in neurodevelopmental disorders, resulting in clinical manifestations. Molecular diagnoses for patients with neurodevelopmental defects are challenging due to phenotypic and genotypic heterogeneity (Fitzgerald, 2015). The molecular diagnostic yield of neurodevelopmental delay, for example, was lower than that of disorders with unique clinical features (Retterer et al., 2016). As a result, it is critical to establish the pathogenic variation spectrum for each relevant gene and establish molecular diagnoses *via* a genotype-driven approach.

However, before this publication, no more than 15 pathogenic *ARFGEF1* variants had been reported in peer-reviewed papers in the ClinVar database, making the interpretation of *ARFGEF1* variants problematic. The mutations result in null alleles, with just one proven pathogenic missense mutation (Thomas et al., 2021) and overlook any additional disease-causing missense mutations. Furthermore, in the study of Thomas et al. (2021), low-penetrance mutations (c.4033C>T and c.3697C>T) of *ARFGEF1* were discovered, and all the preceding mutations in non-Asian populations. More importantly, determining if variants cause a particular disease in a specific gene requires more replication in other unrelated but similarly affected patients. In this study, we searched the local Chigene database for *ARFGEF1* mutations to establish an unquestionable causal link between *ARFGEF1* mutations and neurodevelopmental disorders. This work considerably expands the phenotypic and genotypic range of ARFGEF1-related neurodevelopmental disease by reporting five Chinese cases with uncommon pathogenic/likely pathogenic ARFGEF1 mutations.

## Materials and Methods

The local Chigene database contains whole-exome sequencing (WES) (using IDT xGen Exome Research Panel v1.0/2.0) data from 61,191 probands from 2018 to 2021. All probands had clinical descriptions that included at least one Human Phenotype Ontology (HPO) term. The rare variants in *ARFGEF1* were searched against the Chigene database. Family history, consanguinity, clinical phenotype, and past genetic testing data were extracted from our database. The study was approved by the Ethics Committee of the Children's Hospital, Zhejiang University School of Medicine (reference number 2021-IRB-129). All participants and their parents provided informed consent. Follow-up examinations were performed for all patients until March 2022 (mean follow-up of 10 months). Intellectual disability (ID) was estimated based on The Wechsler Preschool and Primary Scale of Intelligence-IV for Children (WPPSI-IV).

Variants in established diagnostic genes were classified according to the published guidelines of the American College of Medical Genetics and Genomics (ACMG) and the Association for Molecular Pathology (AMP) as pathogenic (P), likely pathogenic (LP), and a variant of unknown significance (VUS) (Richards et al., 2015). Variant annotation was based on *ARFGEF1* transcript NM_006421.4. Sanger sequencing was performed to confirm positive exome findings.

## Results

### Clinical Description

The patient cohort consists of five unrelated individuals from five Chinese families. At the last evaluation, the probands (four boys and one girl) were aged two to 4.5 years. We retrospectively characterized their phenotypes and contrasted them to previously published cases. A summary of the clinical characteristics can be found in Table 1. Except for Proband 3, who experienced hypoxia asphyxia at birth, all individuals were born following uneventful pregnancies and delivered with normal Apgar scores without perinatal complications.

**Table 1:** Clinical features and variants detected in the five probands of the cohort.

	**Proband 1**	**Proband 2**	**Proband 3**	**Proband 4**	**Proband 5**
Age on referral	7 months old	2 years old	2 years old	3 years old	2 years old
Gender	M	M	M	M	F
Birth height	-	50 cm	-	50 cm	-
Birth weight	-	3.4 kg	1.45 kg	3.3 kg	3,550 g
Birth history	G2P2, no abnormal	G1P1, no abnormal	G3P2, 29 weeks of gestation, a history of hypoxia rescue	no abnormal	G1P1, born without asphyxia, the mother has amniotic fluid III degrees pollution
Speech delay	+	+	+	+	+
Motor delay	+	+	+	+	+
Degree of delay	NA	Mild	Moderately severe	Moderate	Severe
Behavioural problems	-	+	ASD	-	-
Intellectual disability (ID)	+	+	+	+	+
Degree of ID	?	Mild	NA	Severe	Severe
Neurological features	-	-	NA	-	-
Neurosensory disorders	-	-	-	Strabismus	-
facial dysmorphisms	-	-	-	-	-
Epilepsy	+	+	+	+	+
MRI findings	-	-	-	-	+
EEG	NA	+?	+	+	+
DST	-	-	-	Testing age: 25 months, Mental age: 7 months for exercise, 8 months for social adjustment, 6 months for intelligence	-
SM	-	-	-	SH: 01, L:01, O: 01, C: 03, S: 02, SD: 00	-
Medication history	levetiracetam	oxcarbazepine, depakin, limbavirin	B vitamins, left-carnitine	-	levetiracetam
Exome sequencing strategy	Trio	Trio	Trio	Trio	Trio
GrCh37/Hg19 genomic variants	g.68163532	g.68116923	g.68200301	g.68140250	g.68152452-g.68152453
cDNA variants: (NM_006421.4)	c.2850+2T>A	c.4951delG	c.917-1G>T	c.3539T>G	c.2923_c.2924delCT
Amino acid variants		p.Ala1651Glnfs^*^24		p.Ile1180Arg	p.Leu975Profs^*^41
Type of mutation	splicing	frameshift	splicing	missense	Frameshift
Familial segregation	*De novo*	*De novo*	Maternally inherited	*De novo*	*De novo*
gnomAD V2.1.1	absent	absent	absent	absent	Absent
dbSNP v153	absent	absent	absent	absent	Absent
ESP6500	absent	absent	absent	absent	Absent
Local data lake	singleton	singleton	singleton	singleton	Singleton
ACMG classification	P(PVS1+PS2+PM2)	P(PVS1+PS2+PM2)	LP(PVS1+PM2)	LP(PS2+PM2+PP3)	P(PVS1+PS2+PM2)

* *Represents the location of the stop codon*.

Clinical deep phenotyping revealed that the most common findings among all patients were: developmental delay (5/5), ID (5/5), and epilepsy (5/5). Although present with variable severity, developmental delay affected speech (5/5) and motor development (5/5). Proband 3 exhibited microcephaly and predisposition for autism spectrum disorders (ASD). Proband 4 had a poor painful feeling and poor hand-eye coordination. These have not been described previously with *ARFGEF1* mutations, and they may be a coincidence. Our cohort did not observe certain previously described features, such as facial dysmorphisms. Brain MRI from proband 5 showed abnormal. The mother of probands 3 showed several features consistent with the *ARFGEF1*-related phenotype, including mild developmental delay and mild ID.

### Molecular Data

During exome analysis, all case subjects' proband-parents' relationships were verified. Five rare disease-causing variants of *ARFGEF1* were identified from our cohort. WES failed to identify any alternative molecular diagnosis potentially causative of the phenotype, excluding family 3, in which we identified an LP variant of *MECP2* in Proband 3 and his affected mother.

**Figure 1:**
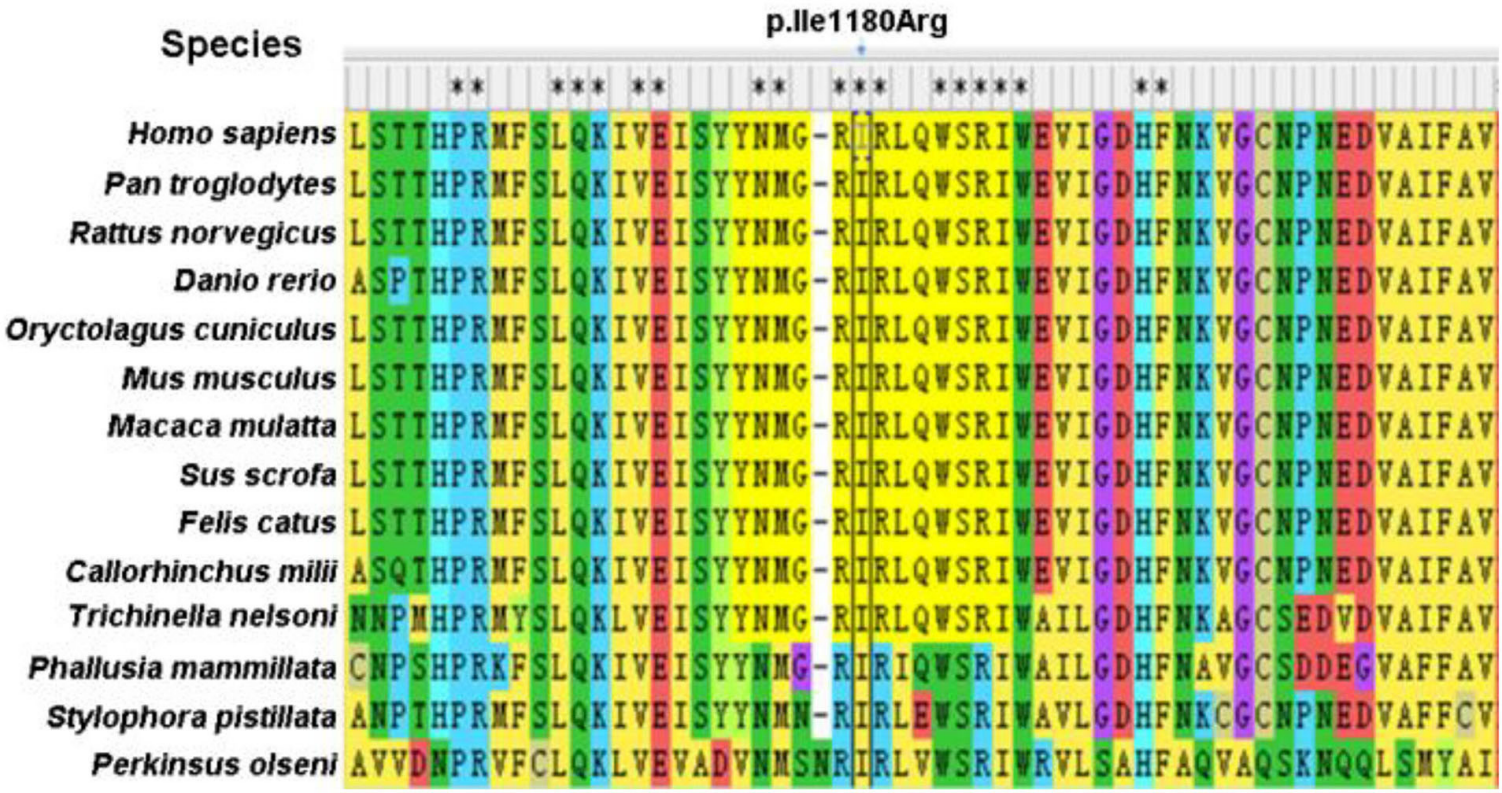
Sequence conservation of the p.Ile1180Arg is indicated in *ARFGEF1* protein among different species.

**Figure 2:**
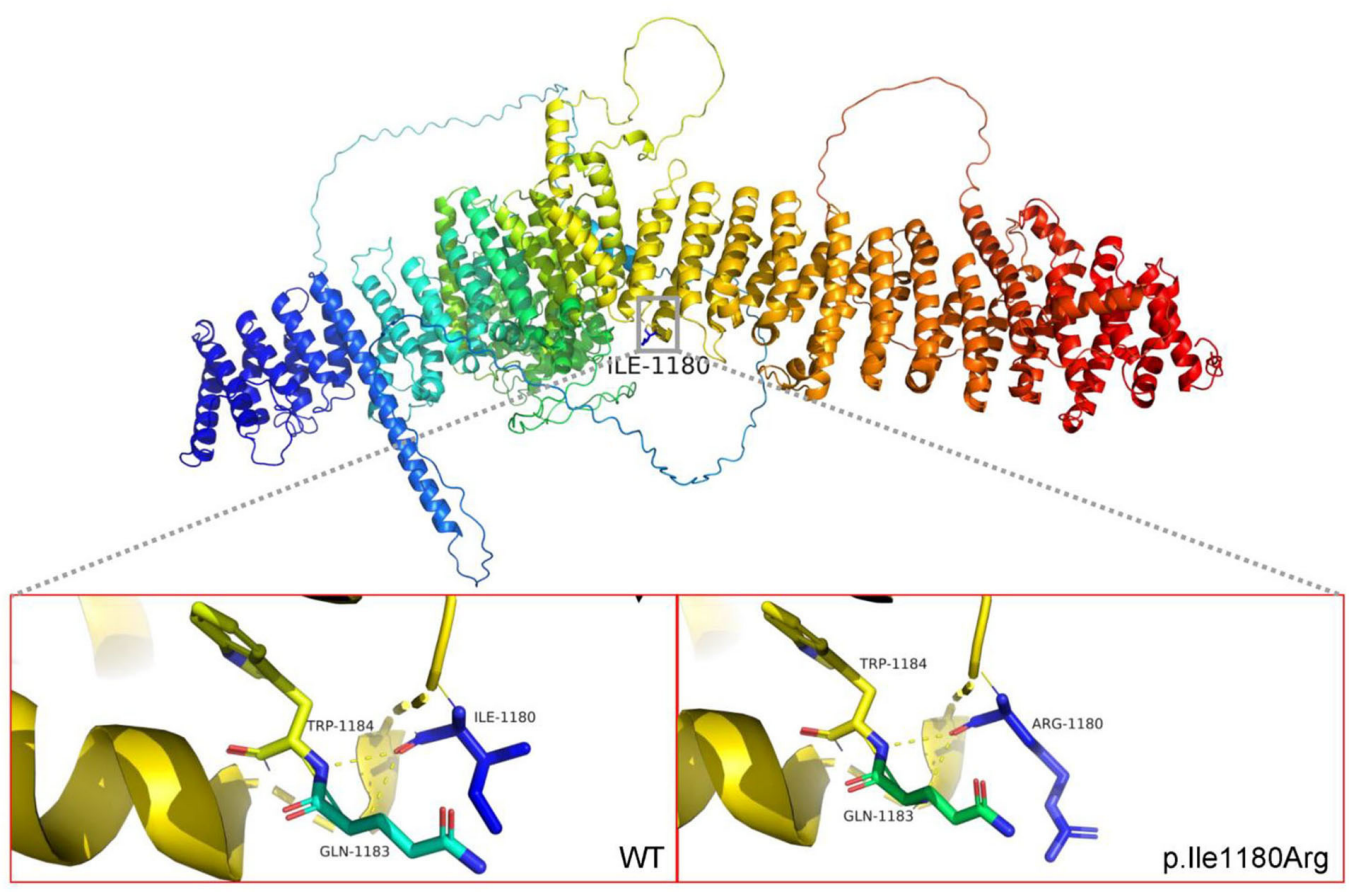
3D protein modelling of *ARFGEF1* missense variants p.Ile 1180 Arg. The amino acid at position 1180 is ILE, located at the HDS2 (homology upstream of Sec7d 2) domain. After mutation to Arg, it changes from non-polar amino acid to polar positively charged amino acid, inducing a change in the local polarity of the protein. The mutation at this position may result in a significant alteration of the HDS2 properties. Protein 3D molecular modeling was predicted by PyMOL (www.pymol.org). Conserved amino acids are represented by “^*^”.

Specifically, Probands 1 and 3 harbor a splicing variant, and Probands 2 and 5 carry a frameshift variant, resulting in a premature stop codon, anticipated to result in protein truncation or to initiate nonsense-mediated decay (NMD) and, thus, loss of the aberrant protein product. Probands 4 was observed to have a missense variant, with multiple *in silico* pathogenicity-prediction tools (Provean, SIFT, Polyphen2, mutation taster, and M-CAP), suggesting a damaging effect. The corresponding p.Ile1180Arg variant affects a highly conserved amino acid residue in the HDS2 domain (Figure 1). This apparent evolutionary conservation as far down as *Perkinsus olseni* implies that this amino acid residue is essential for appropriate protein function. The missense variant's pathogenicity supports 3D molecular modeling, which predicted that the missense variant impairs protein function (Figure 2).

**Figure 3:**
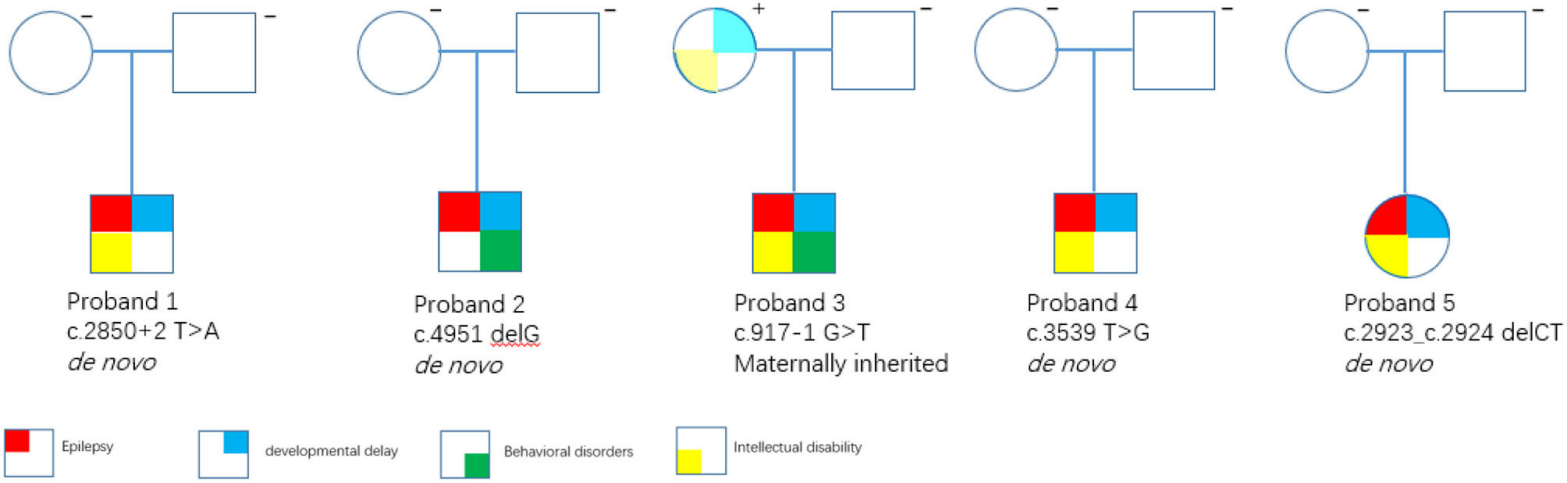
Family trees. Square = male; circle = female; filled symbols = affected individuals; open symbols = unaffected carriers.

All the five variants are rare and absent from the public variant database gnomAD (release v2.1.1), dbSNP, ESP, and only exist as a singleton allele in our local Chigene database. Four patients had *de novo* mutations (probands 1, 2, 4, and 5), and one patient inherited the mutation from his mildly affected mother (probands 3) (Figure 3). The read count data from family 3 did not demonstrate any somatic mosaicism in the blood sample from the affected mother (46/111).

**Figure 4:**
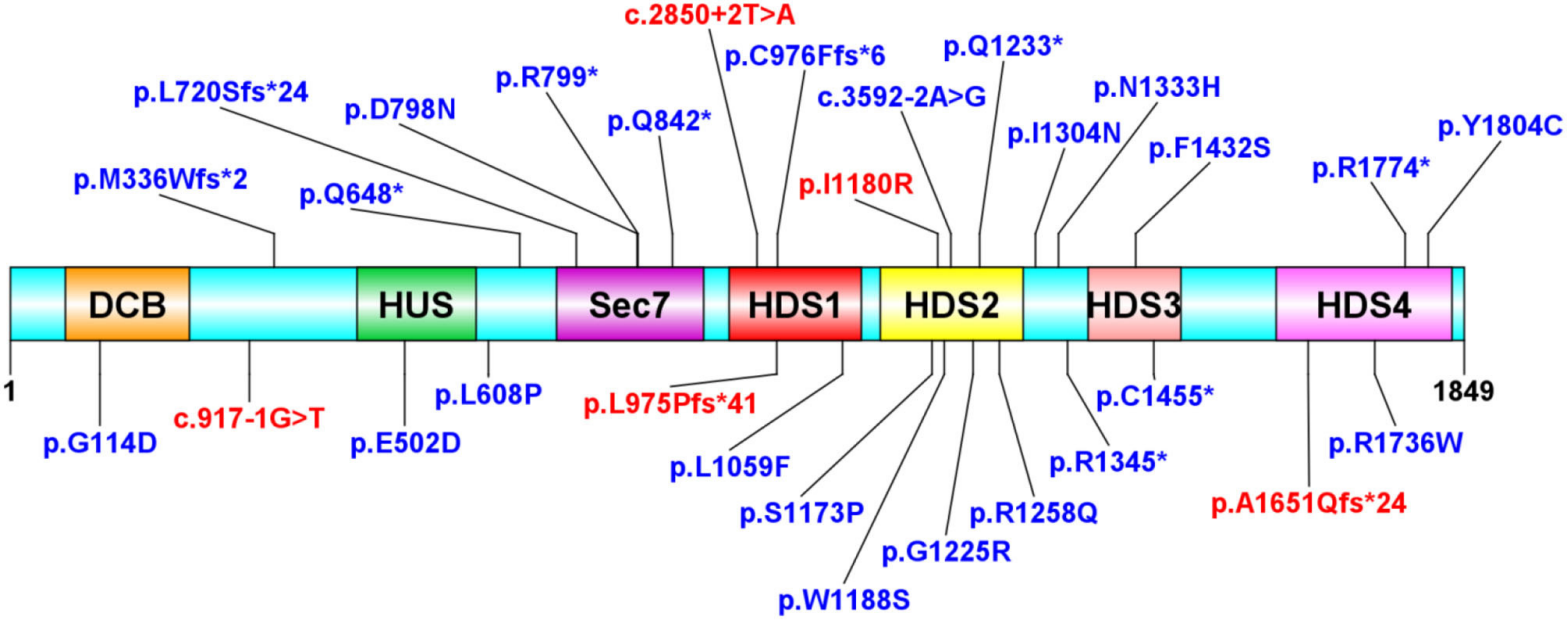
Protein structure distribution of *ARFGEF1* variants. Variants were observed in this study (red). Variants in the literature (blue). The variants are evenly distributed throughout the protein. *Represents the location of the stop codon.

We further surveyed the literature for any (Likely) pathogenic or Variant Uncertain Significance (VUS) heterozygous *ARFGEF1* variants that had been reported at the time this manuscript was prepared (Table 2). Figure 4 depicts a representation of all the protein sequence variants. There were no recurrent variants among the 19 disease-causing mutations observed and noticed that c.2923_2924dup and c.2923_2924del occurred once. Sequence analysis showed that CTCTCT hexanucleotide repeat exists nearby, which may result in slippage mutation.

**Table 2:** Previously reported variant uncertain significance (VUS) variants and pathogenic/likely pathogenic variants in *ARFGEF1*.

**GrCh37/Hg19**	**cDNA variants:**	**Amino acid**	**Type of**	**ACMG**	**ACMG**	**Source**	**Phenotype**
**genomic variants:**	**NM_006421.4**	**variants**	**mutation**	**classification**	**Pathogenicity**		
**chr8**					**basis**		
g.68200211delT	c.1006delA	p.Met336Trpfs^*^2	Frameshift	Likely pathogenic	PVS1+PM2	PMID: 34113008	Neurodevelopmental disorders
g.68178422G>A	c.1942C>T	p.Gln648^*^	Stopgain	Likely pathogenic	PVS1+PM2	PMID: 34113008	Neurodevelopmental disorders
g.68172127delG	c.2158delG	p.Leu720Serfs^*^24	Frameshift	Likely pathogenic	PVS1+PM2	PMID: 34113008	Neurodevelopmental disorders
g.68170369C>T	c.2392G>A	p.Asp798Asn	Missense	pathogenic	PS1+PM1+PM2+PP2+PP3	PMID: 34113008	Neurodevelopmental disorders
g.68170366G>A	c.2395C>T	p.Arg799^*^	Stopgain	Likely pathogenic	PVS1+PM2	PMID: 34113008	Neurodevelopmental disorders
g.68169969G>A	c.2524C>T	p.Gln842^*^	Stopgain	Likely pathogenic	PVS1+PM2	PMID: 34113008	Neurodevelopmental disorders
g.68152452	c.2923_2924dup	p.Cys976Phefs^*^6	Frameshift	Likely pathogenic	PVS1+PM2	PMID: 34113008	Neurodevelopmental disorders
g.68139835T>C	c.3592–2A>G		Splicing	Likely pathogenic	PVS1+PM2	PMID: 34113008	Neurodevelopmental disorders
g.68139728G>A	c.3697C>T	p.Gln1233^*^	Stopgain	Likely pathogenic	PVS1+PM2	PMID: 34113008	neurodevelopmental disorders
g.68138302G>A	c.4033C>T	p.Arg1345^*^	Stopgain	Likely pathogenic	PVS1+PM2	PMID: 34113008	Neurodevelopmental disorders
g.68130347G>T	c.4365C>A	p.Cys1455^*^	Stopgain	Likely pathogenic	PVS1+PM2	PMID: 31678406	Neurodevelopmental disorders
g.68112696G>A	c.5320C>T	p.Arg1774^*^	Stopgain	Likely pathogenic	PVS1+PM2	PMID: 34113008	Neurodevelopmental disorders
g.67266974A>G	c.1823T>C	p.Leu608Pro	Missense	VUS	PM2+PP2+PP3	PMID: 27694994	Schizophrenia
g.67226103T>G	c.3997A>C	p.Asn1333His	Missense	VUS	PM2+PP2+PP3	PMID: 27694994	Schizophrenia
g.67201528G>A	c.5206C>T	p.Arg1736Trp	Missense	VUS	PM2+PP2+PP3	PMID: 27694994	Schizophrenia
g.67299327C>G	c.341G>A	p.Gly114Asp	Missense	VUS	PM2+PP2+PP3	PMID: 27694994	Schizophrenia
g.67227142A>T	c.3911T>A	p.Ile1304Asn	Missense	VUS	PM2+PP2+PP3	PMID: 27694994	Schizophrenia
g.67219474A>G	c.4295T>C	p.Phe1432Ser	Missense	Likely pathogenic	PM1+PM2+PP2+PP3	PMID: 27694994	Schizophrenia
g.67238457G>A	c.3175C>T	p.Leu1059Phe	Missense	Likely pathogenic	PM1+PM2+PP2+PP3	PMID: 27694994	Schizophrenia
g.67227991C>G	c.3563G>C	p.Trp1188Ser	Missense	Likely pathogenic	PM1+PM2+PP2+PP3	PMID: 27694994	Schizophrenia
g.67271768C>G	c.1506G>C	p.Glu502Asp	Missense	Likely pathogenic	PM1+PM2+PP2+PP3	PMID: 27694994	Schizophrenia
g.67199073T>C	c.5411A>G	p.Tyr1804Cys	Missense	VUS	PM2+PP2+PP3	PMID: 27694994	Schizophrenia
g.67227517C>G	c.3673G>A	p.Gly1225Arg	Missense	VUS	PM2+PP2+PP3	PMID: 27694994	Schizophrenia
g.67227280C>G	c.3773G>A	p.Arg1258Gln	Missense	VUS	PM2+PP2+PP3	PMID: 27694994	Schizophrenia
g.67228037A>G	c.3517T>C	p.Ser1173Pro	Missense	Likely pathogenic	PM1+PM2+PP2+PP3	PMID: 28135719	Developmental disorders

* *Represents the location of the stop codon*.

## Discussion

In 2021, Thomas et al. identified an *ARFGEF1*-related neurodevelopmental disorder in a cohort of 13 individuals. Given that gene discovery for conditions with high locus heterogeneity and low-penetrance mutations will necessitate substantially larger sample sizes, we searched our local data lake for information from genetic testing of >60,000 probands. In this article, we reported five new pediatric patients with *ARFGEF1*-related neurodevelopmental disorder and a comprehensive evaluation of the clinical and molecular results in all five cases.

According to evolutionary theory, deleterious alleles are likely to be rare due to purifying selection. For rare missense mutations, pathogenicity becomes stronger, and interpretation of pathogenicity will be challenging. We established the pathogenicity of the missense variant c.3539T>G (p.Ile1180Arg) from multiple perspectives, including frequency in control populations and *in silico* prediction. The missense mutation (p.Ile1180Arg) is a pathogenic mutation, which proves that the pathogenesis of ARFGEF1-related neurodevelopmental disorders may be the loss of function caused by frameshifting, nonsense, splicing, or rare missense mutation of key conserved residues. While this conclusion requires more sample verification.

Males are two to four times as likely than females to acquire neurodevelopmental disorders (May et al., 2019). The authors of the paper by Thomas et al. (2021) noticed an unbalanced sex ratio of the diseases but were unsure if this was due to an actual sex-dependent incidence or in their cohort by chance. We found an uneven sex ratio in our cohort (four males and one female); therefore, we agree with Thomas et al. that the illnesses have a true sex-dependent incidence. Furthermore, we suspect that MECP2 plays a role in establishing imbalanced sex ratios. MECP2 regulates splicing and mRNA template activity and transcription activation and repression (Chahrour et al., 2008; Guy et al., 2011). We detected an *MECP2* missense mutation in proband 5, our cohort's only female patient, but we failed to detect the identical mutation in her asymptomatic mother.

Similarly, we identified an LP of *MECP2* in Proband 3 and his affected mother, who had modest developmental delays. However, we cautioned that this is merely a hypothesis and that more samples will be required to confirm this. In Thomas's work (2021), it is not immediately clear if any harmful *MECP2* mutation occurred in individuals nine (female) and eight and nine's paternal aunt.

Finally, we broadened the genotypic spectrum of *ARFGEF1*-related neurodevelopmental disorder using data from our local Chigene database, identifying five novels heterozygous (likely) pathogenic variants. We provide further evidence of the pathogenicity of haploinsufficiency in male patients and examine the likelihood of digenic inheritance in female patients.

## Data Availability Statement

The datasets presented in this study can be found in online repositories. The names of the repository/repositories and accession number(s) can be found in the article/supplementary material.

## Ethics Statement

The studies involving human participants were reviewed and approved by the Ethics Committee of the Children's Hospital, Zhejiang University School of Medicine. Written informed consent to participate in this study was provided by the participants' legal guardian/next of kin. Written informed consent was obtained from the individual(s), and minor(s)' legal guardian/next of kin, for the publication of any potentially identifiable images or data included in this article.

## Author Contributions

FG, DS, and WG conceived, designed, and supervised the study. LX carried out the statistical analyses. YZ, XR, and CX performed the bibliographic search and analyzed the data. LX wrote the manuscript, with relevant input from FG, DS, and WG. All authors critically revised the article for important intellectual content.

## Funding

This study was supported the Key Research and Development Program of Zhejiang Province (2020C03038).

## Conflict of Interest

CX, SW, and WG were employed by Beijing Chigene Translational Medical Research Centre Co. Ltd. The remaining authors declare that the research was conducted in the absence of any commercial or financial relationships that could be construed as a potential conflict of interest.

## Publisher's Note

All claims expressed in this article are solely those of the authors and do not necessarily represent those of their affiliated organizations, or those of the publisher, the editors and the reviewers. Any product that may be evaluated in this article, or claim that may be made by its manufacturer, is not guaranteed or endorsed by the publisher.
